# A global health model integrating psychological variables involved in cancer through a longitudinal study

**DOI:** 10.3389/fpsyg.2022.873849

**Published:** 2022-07-28

**Authors:** Patricia Macía, Susana Gorbeña, Mercedes Barranco, Nerea Iglesias, Ioseba Iraurgi

**Affiliations:** ^1^Faculty of Psychology, University of Basque Country, UPV/EHU, San Sebastian, Spain; ^2^Department of Psychology, Faculty of Health Sciences, University of Deusto, Bilbao, Spain; ^3^Spanish Association Against Cancer of Biscay, Bilbao, Spain

**Keywords:** cancer, health, stress, coping, psycho-oncology, longitudinal study

## Abstract

**Objective:**

The literature has shown the relevance of certain psychological variables in adjustment to cancer. However, there is a great variability, and these features could be modified through the disease process. The aim of this study is to provide an integrated and global perspective of the importance of variables such as coping, resilience, emotional control, social support, affect, and others in cancer patients through a longitudinal study, with the objective of exploring their associations and underlying interactions.

**Methods:**

The sample was composed of 71 people diagnosed with cancer who were attending psychological support at the Spanish Association Against Cancer (Biscay). We assessed the following variables in two periods of 6 months: perceived stress (PSS), emotional control (CECS), resilience (CD-RISC), coping strategies (CERQ), personality (NEOFFI), social support (MOSS), affect (PANAS), emotional distress (GHQ), quality of life (SF-12) and visual-analogic scales (EVA).

**Results:**

Results showed predictive effects of perceived stress on physical health perception (*β* = −0.22; *t* = −3.26; *p* = 0.002). Mental health perception was influenced by almost all the psychological variables. Consciousness at baseline (*β_Co_* = 0.15; *p* = 0.003), change in Extraversion (*β_Ex_* = 0.16; *p* = 0.001) and Resilience (*β_Re_* = 0.15; *p* = 0.002) had significant effects on perceived mental health.

**Conclusion:**

This study provides a global health model that integrates and explores associations between psychological variables related to cancer disease. This information could be useful for guiding personalized psychotherapeutic interventions, with the aim of increasing adjustment to disease.

## Background

People suffering from cancer disease are exposed to high levels of stress that influence their level of mental health and well-being. Cancer is considered a life-threatening disease that can provoke reactions of fear and vulnerability (Cordova et al., 2017). Besides, the characteristics of the oncological treatment can also lead to physical and psychological consequences. In fact, people under oncological treatment report more level of chronic fatigue, limited physical roles, and even higher emotional distress (Aarts et al., 2015; Merluzzi et al., 2018). Cancer patients frequently complain not only about bodily pain and fatigue, but also about symptoms of emotional distress (Seib et al., 2018). For instance, it has been documented that people with cancer experience anxiety and depression levels to a larger extent than the general population (Moreno et al., 2015; Hernández and Cruzado, 2016).

However, reactions and adaptation of each individual to disease can vary from one person to another, and even among different stages of the disease (Deimling et al., 2006). In this regard, research on psychological variables has acquired relevance for explaining differences in adaptation to cancer. For instance, fear of cancer is a frequent emotion both in patients with cancer and survivors. Specifically, fear of cancer recurrence (FCR) has been investigated in relation to psychological intervention focused on adapting to the health process. Cognitive behavioral therapies (CBTs) have been used in clinical practice with oncological patients (Cincidda et al., 2022). In this sense, psychological variables would adopt a state characterization, with the purpose of modifying certain coping ways and/or mental states for raising a better well-being and quality of life along the cancer process.

In this respect, variables such as coping strategies and resilience have been investigated in oncological patients (Lai et al., 2020). Coping refers to thoughts and behaviors to deal with the demands of stressful and adverse events, as can be cancer disease (Lazarus and Folkman, 1984). People use different coping strategies depending on the situation, and two types of strategies have been distinguished: adaptive and disadaptive. Adaptive coping has been related to positive mental health outcomes in cancer (Roesch et al., 2005; Lim et al., 2014), while disadaptive coping strategies (such as rumination, self-blame and suppression) have proved to be linked to poorer physical and psychological health (Nipp et al., 2016; Lai et al., 2020).

On the other hand, resilience has been conceived as the ability to cope effectively with stressful or traumatic situations, emerging even stronger and maintaining mental health (Masten, 2001; Bonanno, 2012). It has been connected to positive emotional health outcomes in cancer. Resilience has been linked to the use of active and adaptive coping styles (Smith et al., 2016; Macía et al., 2020a). It has demonstrated to be a protective factor for emotional health, consequently leading to a better accommodation to cancer (Lai et al., 2020).

Another concept investigated in relation to adjustment to cancer is emotional control. It has been defined as the attempts to avoid or control the expression of negative feelings (Anarte et al., 2001). Emotional control, in contrast to emotional expression, has been associated with psychosocial maladjustment in cancer patients (Durá et al., 2010; Lemogne et al., 2013). In fact, suppression of certain emotions has been related to higher symptoms of depression in cancer patients undergoing chemotherapy (Schlatter and Cameron, 2010). Furthermore, emotional control has been considered as the main characterization of the Type C personality, which has been linked to cancer onset and prognosis (Greer and Watson, 1985; Gross, 1989; Durá et al., 2010; Li et al., 2015). Type C pattern is defined as a type of personality characterized by lack of assertiveness, conformity, patience and the inexpression of negative emotions, such as anger and concern (Durá et al., 2010; Li et al., 2015). Conversely, emotional expression and acknowledgement of traumatic situations have demonstrated to be connected to physical and psychological health (Pennebaker et al., 1997; Pennebaker, 2000).

There are also certain personality traits that been investigated in relation to mental health in cancer. For instance, neuroticism and extraversion might increase cancer risk according to psychosomatic theories (Kissen and Eysenck, 1962). Specifically, the Five-Factor Model (Costa and McCrae, 1985) has been widely used for defining personality dimensions and involves extraversion, conscientiousness, agreeableness, neuroticism and openness to experience. Precisely, neuroticism, extraversion and consciousness have been assessed features in relation to cancer. Extraversion has been linked to positive mental health (Hoerger et al., 2016; You et al., 2018), while neuroticism has been associated with poorer health levels, accompanied by physical symptoms such as pain, among others (Krok and Baker, 2014). Besides, consciousness has also showed correlations with mental health in people with cancer (Bogg and Roberts, 2013). In brief, some personality traits (such as neuroticism) characterized by the use of less adaptive coping and risk of developing distress seem to be risk factors for mental health. While other features related to openness and seeking for social support (such as extraversion, for instance) are more related to positive health outcomes (You et al., 2018).

In this respect, social support is another significant aspect regarding adaptation to cancer. Numerous investigations have shown its protective effects on health, and particularly, when it is a serious physical illness such as cancer (Applebaum et al., 2014; Vinaccia et al., 2014). It has been related to adequate psychological adjustment in cancer (Mishra and Saranath, 2019). Nevertheless, a decrease in quality and quantity of social support predicts higher stress and more depressive symptoms in patients (Fong et al., 2017). However, perceiving significant social support contributes to healthy behaviors, both in the biomedical and psychosocial sphere (Costa-Requena et al., 2014). Furthermore, it has also been associated with more positive affect, which is other important variable in cancer research.

Indeed, affect has been commonly assessed in cancer patients due to its association or influence on health. It refers to the emotional state or disposition that encompasses emotional responses and/or fluctuations when dealing with different situations (Watson et al., 1988). Affect is measured in terms of balance of affect, which involves two polarities: positive and negative affect. Specifically, negative affect has been related to poorer health outcomes, with predominance of psychological symptoms of distress in cancer patients, such as depression and anxiety (Watson et al., 1988; Li et al., 2018). Conversely, positive affect, often characterized by feelings of pleasure and satisfaction, has been linked to well-being and positive health outcomes in cancer. In fact, it has shown a mediation effect on the relation between resilience and physical symptoms in people with cancer, leading to a decrease in disease-related fatigue (Zou et al., 2018).

The literature has shown the relevance of psychological variables in adjustment to cancer. It is noteworthy that certain psychological features and resources contribute to better adaptation to disease. However, the presence of these aspects shows a great variability and can be modified through the disease process. Although many of these factors have been previously investigated in cancer patients, few studies have integrated all those variables and have research on their associations and underlying interactions through a longitudinal study. The aim of this study is precisely to provide an integrated and global perspective of the importance of psychological variables in cancer patients. This could be relevant information for guiding the design of therapeutic interventions tailored and personalized to each person’s reality. The main objective of this study is to explore the predictive effects of psychological variables on health perception in people with cancer. Considering the existing literature and clinical experience in psycho-oncology, we hypothesize that: (1) participants will show poor health perception levels; (2) after 6 months their health perception would be worsened; (3) also, stress and emotional distress would be increased after 6 months, affecting both physical and mental health; (4) psychological variables such as resilience, adaptive coping, social support and certain personality traits would show a positive predictive effect on mental health perception in people with cancer; (5) however, emotional control would show a negative impact on health perception.

## Materials and methods

### Participants

The sample was composed by 71 people with cancer. They came from the Spanish Association Against Cancer (AECC) of Biscay where they receive support and/or counseling services provided by licensed health care professionals. Ages of the participants ranged from 37 to 74 years old (*M* = 53.03, *SD* = 8.16) and 83.1% of them were women. Sociodemographic characteristics of the participants are shown in Table 1.

**Table 1:** Sociodemographic and clinical variables for the oncological sample (*n* = 71).

Sociodemographic variables		*n*	%		Clinical variables		*n*	%
Gender	Female	59	83.1		Stages	I	3	4.2
	Male	12	16.9			II	7	9.9
Ed. Level	Primary school	5	7.0			III	10	14.1
	Secondary school	13	18.3			IV	30	42.3
	Professional training	16	22.5					
	University	36	50.7					
	Others	1	1.4					
Employment	Paid work	34	47.9		Oncological treatment	Yes	64	92.8
	Unpaid work	0	0.0			No	5	7.2
	Unemployed	3	4.2					
	Retired	11	15.5					
	Disabled	22	31.0					
	Others	1	1.4					
Civil status	Single	10	14.1		Other medical treatment	Yes	37	58.7
	Married, in couple	53	74.6			No	26	41.3
	Separated, divorced	7	9.9					
	Widower	1	1.4					
	Others	0	0.0					

*n*, sample size; %, percentage.

All the participants had been diagnosed with cancer [breast (46.5%), lung (7%), colon (11.3%), gynecological (14.4%), prostate (2.8%), pancreas (1.4%), bladder (2.8), among others (12.7%)]. Almost all of them (92.8%) had received oncological treatment (chemotherapy and radiotherapy) and other medical treatments (58.7%). Regarding the stage of the disease, 56.4% of the participants were in advanced phases (stages III and IV). A 91.4% had received or were receiving psychological assistance more or less frequently at AECC. Inclusion criteria to participate in the study were: to be over 18 years old, to suffer or have suffered from cancer diagnosis, and to be contact of the AECC. Conversely, exclusion criteria were to be under 18 years old and to have never been diagnosed with cancer.

### Procedure

All the participants who were attending the association, or at least had a close relation with it, were asked to participate voluntarily in the study. Data was collected by psychologists from the Spanish Association Against Cancer of Biscay with wide experience in cancer patient care. Participants were provided with information about the study by email or at the association. In case affirmative, a written informed consent was obtained before data collection in order to meet all ethical and legal requirements of the research project. All participants completed a self-administered questionnaire (see Instruments) twice, with a period of 6 months between each evaluation. The questionnaire could be answered in paper (at the premises of the association) or online, as best suited them. It took about 50 min completing the questionnaire, approximately. If any emotional reactions emerged, the psychologists of the AECC committed to maintain an empathic attitude, providing support.

### Instruments

A self-administered questionnaire was used to collect data, including socio-demographical and clinical information about the disease. Psychometric instruments were used to analyze the variables of interest in people with cancer. It took about 50 min approximately to fill the entire questionnaire.

#### Physical and emotional health

##### Quality of life

Perception of quality of life was evaluated with the General Health Questionnaire SF-12 (Ware et al., 1996), based on the SF-36 (Ware and Sherbourne, 1992). It was adapted into Spanish version (Alonso et al., 1995), showing good internal consistency levels over *α* = 0.70 in all subscales. The questionnaire assesses eight dimensions: Physical Functioning (PF), Role limitations due to Physical health problems (RP), Social Functioning (SF), Bodily Pain (BP), Mental Health (MH), Role limitations due to Emotional problems (RE), Vitality (VI) and General Health (GH). The instrument presents good internal consistency, with a Cronbach’s alpha of.77. The overall score is obtained by summing the scores, evaluated through a Likert scale. The instrument provides two total scores or components (mental and physical health–TMC (*α* = 0.91), and TPC (*α* = 0.91) respectively), which are expressed in standardized T scores. These scores were obtained through the application of specific algorithms, which were provided by the group of people that adapted the instrument in Spain, under the direction of the Municipal Institute of Medical Research of Barcelona.

##### Emotional distress

The 12-item General Health Questionnaire—GHQ-12 was selected to assess level of emotional distress or mental health (Goldberg and Hillier, 1979; Lobo et al., 1986; Sánchez-López and Dresch, 2008). The questionnaire was developed to detect diagnosable psychiatric disorders. It is intended for adults who have to indicate the frequency with which they have experienced some symptoms. The scale was measured on a Likert scale with four response options ranging from “0 = better than usual” to “3 = much worse than usual.” It showed good internal reliability, with a Cronbach’s Alpha for the Spanish version of *α* = 0.76, and *α* = 0.93 for this study.

##### Visual analog scale (EVA)

Eight visual scales were used to assess subjective perception of health and satisfaction with treatment through a series of thermometers (EVA). They were originally developed for assessing pain intensity, represented by a 10 cm line with two extremes: “0 = no pain” and “10 = the worst pain imaginable.” The point in the line marked by the participant represents the intensity of the pain (Bijur et al., 2001). In this research this measure has been extrapolated to other measures of satisfaction and well-being, as it is a reliable and valid scale for assessing different issues related to health estimation. The range of scores for each response was between 0 and 10.

#### Perceived stress

Perceived stress was assessed with the 10-item Cohen Perceived Stress Scale (PSS; Cohen et al., 1983). It consist of a five-point Likert scale with response options ranging from “0 = never” to “4 = most often.” The scale shows a factorial dimensionality with two main factors: stress control and no control. Internal consistency for the original study is *α* = 0.75 (Cohen et al., 1983). The Spanish adaptation by Remor (2006) obtained a similar factorial structure and a reliability of *α* = 0.81. Cronbach’s Alpha for this study was *α* = 0.93 for the total score, *α* = 0.86 for stress control subscale and *α* = 0.88 for no-control subscale.

#### Resilience

Resilience was measured with the 10-item Connor-Davidson Resilience Scale-CD-RISC (Connor and Davidson, 2003; Campbell-Sills and Stein, 2007). The items presented a five-point Likert scale, ranging from “1 = totally disagree” to “5 = totally agree.” The total of resilience was obtained summing the items, so that higher scores indicated higher resilience. The scale showed appropriate psychometric qualities, with a Cronbach’s alpha of *α* = 0.85 in the original study, *α* = 0.81 in the Spanish version of 10 items (Notario et al., 2011; Serrano-Parra et al., 2013) and *α* = 0.92 in the present study.

#### Emotional control

The Courtauld Scale of Emotional Control-CECS (Watson and Greer, 1983) was selected to assess emotional control. It was adapted to Spanish by Anarte et al. (2001). It assesses the underlying construct of Type C behavior pattern to analyze to what extent individuals try to control their emotional expression when experiencing negative emotions. The 21 items are measured on a four-point Likert scale, ranging from “1 = almost never” to “4 = almost always.” Items are divided into three subscales (Brandão et al., 2016): anger, anxiety, and depressed-mood. Cronbach’s alpha in the original study and the Spanish version of the scale (into parenthesis) was.88 (0.94) for the total, 0.86 (0.92) for anger, 0.88 (0.93) for depressed-mood, and.88 (0.93) for anxiety. Cronbach’s alpha in this study was *α* = 0.92 for the total, *α* = 0.87 for anger, *α* = 81. for depressed-mood, and *α* = 0.90 for anxiety.

#### Coping strategies

The Cognitive Emotion Regulation Questionnaire-Short Scale (CERQ; Garnefski and Kraaij, 2006) was used to assess coping strategies. It evaluates the cognitive assessment when facing stressful life events. The 18 items are divided into nine subscales that were conceptually grouped in two main dimensions: adaptive (acceptance, positive refocusing, planning, positive reappraisal and put into perspective) and disadaptive coping (self-blame, rumination, catastrophizing and blame others). The scale was adapted to Spanish version (Galego, 2016). The scale presents a five-point Likert scale ranging from “1 = hardly ever or never” to “5 = almost always.” The original study showed good psychometric qualities, with Cronbach’s alpha coefficients over.80, and good factorial, discriminative and construct validity (Garnefski and Kraaij, 2006). Cronbach’s alpha in this study was *α* = 0.83 for adaptive coping and *α* = 0.73 for disadaptive coping.

#### Affect

Positive and Negative Affect Schedule-PANAS (Watson et al., 1988) has been selected to measure the affect of participants. It shows a bifactorial structure, formed by a Positive Affect (PA) scale and a Negative Affect (NA) scale, with 10 items each one. It consists of a five-point Likert scale, ranging from “1 = nothing or very slightly” to “5 = a lot.” The total of the scale is obtained summing all scores, so that higher score means higher affect of that type. The scale has been adapted to Spanish population (López-Gómez et al., 2015). It shows good psychometric qualities, with a Cronbach’s Alpha of.92 for the Positive Affect scale and.88 for the Negative Affect scale. Cronbach’s alpha in this study is *α* = 0.74 for the balance of affect, *α* = 0.84 for the positive affect and *α* = 0.91 for the negative affect.

#### Perceived social support

To evaluate social support the Social Medical Outcomes Study—Social Support Survey (MOS-SSS; Sherbourne and Stewart, 1991) was selected. It is formed by 19 items that are grouped into 4 dimensions: emotional/informational support, positive social interaction, emotional support and instrumental support. They are measured through a five-point Likert scale with response options ranging from “1 = never” to “5 = always.” The total sum of all dimensions provides the Global Social Support Index. The scale has been adapted to a Spanish cancer population, showing a Cronbach’s alpha of *α* = 0.94 (Requena et al., 2007). Internal consistency in this study was *α* = 0.96 for the total of the scale.

#### Personality: Neuroticism, extraversion and consciousness

The 60-item NEO Five-Factor Inventory (NEO-FFI; Costa and McCrae, 1985) was developed to measure the five basic personality traits. Each subscale has 12 items, which were selected from the initial pool of 180-item NEO Personality Inventory (NEO-PI; Costa and McCrae, 1992). The instrument has a five-points Likert scale, with options ranging from “0 = total disagreement” to “4 = strongly agree*.”* The direct score on each scale is obtained by adding up the subjects’ responses to the corresponding items. The NEO-FFI has been translated into several languages and has shown validity and replicability in many different contexts. The Spanish adapted version presents good internal consistency with the following Cronbach’s alpha: neuroticism (0.82), extraversion (0.81), openness (0.76), agreeableness (0.71) and conscientiousness (0.81; Manga et al., 2004; McCrae and Costa, 2004). Internal consistency for this study was neuroticism.85, extraversion.85, openness 0.76, agreeableness 0.69 and conscientiousness.84. For this study, three specific dimensions were selected from the NEO-FFI due to their specific relation with mental health in cancer patients: neuroticism, extraversion and consciousness (Bogg and Roberts, 2013; Chapman et al., 2014; Perry et al., 2018).

### Statistical analyses

Firstly, descriptive statistics (*M* and *SD*) were calculated for the variables of interest in both evaluation moments (at the baseline and the follow-up), and also the change score was obtained. To facilitate a better comprehension, all measures were transformed to a decimal scale. Correlations (Pearson’s *r*) and Student *t* were performed to explore if statistical significant differences existed between both periods. Furthermore, effect size (Hedges’ *g*) was calculated to estimate the size of the differences between both times. Additionally, Cronbach’s Alpha for each scale was obtained, to test the internal reliability (see Instruments). SPSS software version 22 was used to perform these statistical analyses (SPSS I, 2013).

Secondly, a hierarchical regression model was performed for each of the variables. The aim was to explore the predictive effect of psychological variables (stress, resilience, emotional control, adaptive and disadaptive coping strategies, affect, social support and personality) on physical and emotional health (as outcome variables). For this, both physical and mental health (as two global indicators) were analyzed through three components separately: emotional distress (GHQ-12), mental and physical component of quality of life (TMC, TPC) and five visual scales (EVA).

Thirdly, considering the significant relations between variables with health indicators, a path analysis was performed through a structural equation model (SEM) to test the effects between the constructs, estimated with the EQS 6.1.27 (Bentler, 2004). For this purpose, an Exploratory Factor Analysis (AFE) was conducted in order to asses and establish a single Health Status indicator in both evaluation periods: Health Outcome T1 and Health Outcome T2. The model was computed to test the relationships and to represent them graphically. An analysis of the measurement model would indicate if the observed variables measured the latent constructs. Adequate indexes in an initial estimation of the structural model would justify the existence of a conceptual relationship among the different dimensions. To assess the plausibility of the structural equation model, different fit criteria were used (Hu and Bentler, 1999): (a) the Chi-Square statistic (*χ^2^*); (b) a Comparative Fit Index (CFI); (c) the Goodness-of-Fit Index (GFI) as a measure of proportion of variance or covariance explained through the model,; (d) the Adjusted Goodness-of-Fit Index (AGFI); and (e) a Root Mean Squared Error of Approximation (RMSEA). The model was computed assuming that each observed variable was significantly contributing to its respective latent variable.

## Results

Table 2 presents baseline and post-test data for variables of interest. Likewise, change scores (*M_dif_*), contrast of differences between both time periods and effect size (*g*) of the observed change are presented. Scores are expressed in a decimal scale (0–10), where a higher score would indicate a greater expression of the valued construct.

**Table 2:** Mean differences in scores between Time 1 and Time 2 for the oncological sample.

	Baseline		Follow-up		Change		Statistical tests			
	M	SD	M	SD	M_dif_	SD_dif_	*r*	*t*	*p*	*g*
GHQ—Mental Health	4.00	1.99	3.83	1.84	−0.17	1.50	0.70	−0.95	0.347	0.09
**SF-12—quality of life**										
Physical functioning	5.88	3.41	6.09	3.32	0.21	2.45	0.73	0.73	0.471	0.06
Physical rol limitation	5.72	3.19	6.21	3.00	0.49	2.21	0.75	1.88	0.064	0.16
Body pain	7.08	3.08	7.08	3.08	0.00	2.28	0.73	0.00	1.00	0.00
General health	4.72	2.24	4.80	2.45	0.08	1.82	0.70	0.39	0.697	0.03
Vitality	4.93	2.70	5.14	2.53	0.21	2.42	0.58	0.74	0.464	0.08
Social functioning	6.97	2.70	7.68	2.44	0.70	2.32	0.60	2.56	0.013	0.27
Emotional rol limitation	7.06	2.53	7.55	2.22	0.49	0.25	0.63	2.01	0.048	0.20
Mental health	6.06	2.00	6.21	1.90	0.16	1.74	0.60	0.77	0.446	0.08
*Summary Physical H.*	4.16	1.22	4.21	1.20	0.05	0.76	0.80	0.59	0.558	0.04
*Summary Mental H.*	4.38	1.11	4.57	1.03	0.19	0.88	0.66	1.80	0.077	0.18
**Health perception (EVA)**										
Last week	6.64	1.84	6.65	1.88	0.01	1.74	0.56	0.02	0.986	0.01
Last month	6.26	1.92	6.70	1.83	0.45	1.62	0.63	2.33	0.023	0.24
Quality of life	7.17	1.84	7.14	1.68	−0.03	1.58	0.60	−0.15	0.881	0.02
Activity management	7.47	1.95	7.39	1.93	−0.07	1.52	0.69	−0.41	0.684	0.04
Emotional well-being	6.58	2.38	6.59	1.98	0.01	1.91	0.63	0.05	0.963	0.01
**Resilience**	6.42	1.73	6.05	1.71	−0.38	1.28	0.72	−2.48	0.016	0.21
**Emotional control**										
Total	4.48	1.91	4.39	1.78	−0.09	1.18	0.80	−0.63	0.532	0.05
Anger	4.26	2.49	3.90	2.34	−0.35	1.95	0.68	−1.48	0.145	0.14
Sadness	4.54	2.05	4.44	1.92	−0.10	1.52	0.71	−0.54	0.593	0.05
Anxiety	4.63	2.08	4.83	2.10	0.21	1.31	0.80	1.28	0.204	0.10
**Coping**										
Acceptance	7.22	2.90	6.88	3.15	−0.33	3.41	0.37	−0.83	0.412	0.11
ConcOthers	5.42	2.72	5.18	2.83	−0.25	2.78	0.50	−0.75	0.457	0.09
Planning	5.18	2.65	4.60	2.78	−0.58	2.99	0.39	−1.64	0.107	0.21
Positive revaluation	6.21	3.15	5.93	3.03	−0.28	2.67	0.63	−0.89	0.376	0.09
Perspective	4.96	3.20	4.31	2.86	−0.65	2.69	0.61	−2.04	0.045	0.21
*Adaptive*	5.80	2.00	5.38	1.93	−0.42	1.77	0.60	−1.99	0.050	0.21
Self blame	1.48	1.87	1.37	1.76	−0.11	1.92	0.44	−0.46	0.645	0.06
Rumination	3.87	2.78	3.84	2.67	−0.04	2.22	0.67	−0.13	0.894	0.01
Catastrophizing	2.09	2.70	2.22	2.33	0.12	2.79	0.39	0.37	0.711	0.05
Blameothers	0.44	1.22	0.37	0.88	−0.07	0.87	0.70	−0.68	0.497	0.06
*Disadaptive*	1.97	1.57	1.95	1.33	−0.02	1.24	0.65	−0.15	0.881	0.01
**Stress**										
Total	4.06	2.03	3.92	1.88	−0.14	1.34	0.77	−0.89	0.378	0.07
Control	3.64	1.98	3.82	1.92	0.18	1.40	0.74	1.10	0.274	0.09
No control	4.48	2.32	4.01	2.01	−0.46	1.64	0.72	−2.40	0.019	0.21
**Affect**										
Balance of affects	1.88	2.67	2.07	2.44	0.19	1.97	0.71	0.80	.427	0.07
Positive affect	4.47	1.63	4.39	1.43	−0.08	1.39	0.65	−0.51	.610	0.05
Negative affect	2.60	1.89	2.33	1.61	−0.27	1.72	0.53	−1.31	.195	0.15
**MOS—social support**										
Total	8.03	1.92	7.80	1.89	−0.22	1.35	0.75	−1.39	0.171	0.12
Emotional-informational	7.58	2.16	7.49	2.06	−0.09	1.70	0.68	−0.46	0.649	0.04
Instrumental	8.02	2.42	7.76	2.65	−0.26	1.68	0.79	−1.28	0.203	0.10
Social	8.06	2.05	7.71	1.96	−0.35	1.46	0.74	−2.03	0.046	0.17
Affective	8.44	2.24	8.25	2.21	−0.19	1.59	0.75	−1.00	0.323	0.08
**NEOFFI—personality**										
Neuroticism	4.73	2.08	4.48	1.87	−0.25	1.21	0.82	−1.76	0.082	0.12
Extraversion	6.21	1.55	6.07	1.67	−0.13	1.15	0.75	−0.99	0.328	0.08
Openness	6.32	1.48	6.18	1.48	−0.14	0.99	0.78	−1.18	0.242	0.09
Amability	6.70	1.09	6.53	1.20	−0.16	0.95	0.66	−1.43	0.156	0.14
Consciousness	6.47	1.74	6.60	1.59	0.13	0.95	0.84	1.16	0.252	0.08

M, mean; SD, standard deviation; r, Pearson r correlation; *t*, Student *t*; *p*, significance level; g, Hedge’s *g*.

### Health status at baseline

In the absence of data from a healthy normative group that would act as a reference to assess deviation of the analyzed group scores-people affected by an oncological disease-, the observed average value will be taken as a reference with respect to its position in the decimal range, in which health indicators have been homogenized. That is, the value “5” would be the most equidistant point between the possible extreme values (0 and 10); so that scores below 5 would indicate low (*x* ≤ 2.5) or moderate intensity (2.5 < *x* < 5) of the assessed phenomenon, and above value 5 moderate-high (5 ≤ *x* < 7.5) or high (*x* ≥ 7.5) intensity. Assuming this criteria, it is observed that average values in health variables at baseline (Table 2) reflect a moderate or adequate level of health perception in participants: values below 5 in GHQ (*M* = 4) indicate an expression of emotional symptoms of moderate-low intensity, as well as in the SF-12 summary components of physical (*M* = 4.16) and mental health (*M* = 4.38); and in the dimensions of general health (*M* = 4.72) and vitality (*M* = 4.99), showing a compromised health-related quality of life.

However, it should be noted that mean scores of other dimensions of SF-12 (5.88 < *M* < 7.08) and of the assessment of health perception (6.26 < *M* < 7.47) are within a range of moderate-high scores, indicating an adequate health expression. In general, indicators of psychological variables also show positive values: a moderate-high level of resilience (*M* = 6.42), a positive affects balance (*M* = 1.88), a high perception of social support (*M* = 8.03), a greater expression of adaptive coping (*M* = 5.80) than disadaptive (*M* = 1.97), an adequate level of emotional control (*M* = 4.48), a moderate-low stress (*M* = 4.06), and lower expression of neuroticism (*M* = 4.73) than other more adaptive dimensions of NEOFFI (from a minimum of 6.21 in extraversion to a maximum of 6.70 in amability).

### Assessment of change

In general, change scores indicate an improvement in health perception. For instance, positive *M_dif_* scores in the SF-12 summary components of physical (0.05) and mental health (0.19), as well as the balance of affect (0.19), would indicate an increase in quality of life perception and in positive affect; while negative scores in emotional distress (GHQ = −0.17), stress (−0.14) or neuroticism (−0.25) would indicate a decrease in the intensity of these constructs. However, of the 48 analyzed outcome variables, only eight are statistically significant (*p* < 0.05) and three are close to significance (0.05 < *p* < 0.10). There are not even moderate effect sizes in no case (*g* < 0.30). Overall, and given the number of multiple comparisons, the application of Bonferroni’s criteria would imply the rejection of the hypothesis of relevant change differences between pre-/post-test, assuming that the differences found are due to chance.

### Analysis of the predictive effect of psychological variables on physical and emotional health

The second and third objectives of this study consist of identifying which psychological indicators had a predictive role in the change of health perception, considering the following outcome variables: summary components of SF-12 regarding quality of life related to physical health (objective 2) and mental health (objective 3). Table 3 presents the set of stepwise regressions conducted on each of the nine psychological indicators, which shows significant association with physical health at baseline. Given that the best predictor of physical health in the post-test is physical health perception in the pre-test (*r* = 0.84, *p* < 0.001), this indicator has been introduced in Step 1 of the set of regression models, to subsequently assess the change due to the influence of this indicator at baseline (Step 2) and/or the influence of the change in that indicator (Variable_Dif) during the study (Step 3).

**Table 3:** Regressions with physical health.

	Step 1		Step 2		Step 3			
	β	*t*	β	*t*	β	*t*	F	∆R^2^
**Stress**								
PhysicalHealth_T1	0.84	13.01^*^	0.77	9.67*	0.72	9.38^*^	169.28	0.710
Stress_T1			−0.11	−1.37	−0.24	−2.83^*^	86.65	0.008
Stress_Dif					−0.22	−3.26^*^	69.51	0.039
**Resilience**								
PhysicalHealth_T1	0.84	13.01^*^	0.83	12.06^*^	0.84	12.22^*^	169.28	0.710
Resilience_T1			0.04	0.53	0.08	1.04	83.90	0.001
Resilience_Dif					0.11	1.50	57.70	0.009
**Emotional control**								
PhysicalHealth_T1	0.86	13.43^*^	0.86	12.64^*^	0.87	12.72^*^	180.34	0.738
Emotional Control_T1			−0.01	−0.03	0.03	0.45	88.77	0.000
Emotional Control_Dif					0.08	1.15	59.91	0.005
**Social support**								
PhysicalHealth_T1	0.84	13.01^*^	0.83	11.95^*^	0.83	11.72^*^	169.28	0.710
Social Support_T1			0.05	0.76	0.05	0.70	84.42	0.002
Social Support_Dif					−0.04	−0.06	55.45	0.000
**Adaptive coping**								
PhysicalHealth_T1	0.84	13.01^*^	0.84	12.84^*^	0.86	13.50^*^	169.28	0.710
Adaptive_T1			0.01	0.15	0.10	1.32	83.45	0.000
Adaptive_Dif					0.18	2.49	61.98	0.025
**Disadaptive coping**								
PhysicalHealth_T1	0.84	13.01^*^	0.83	11.92^*^	0.82	11.65	169.28	0.710
Disadaptive_T1			−0.03	−0.40	−0.08	−0.92	83.69	0.001
Disadaptive_Dif					−0.08	−1.02	56.18	0.004
**NEOFFI-neuroticism**								
PhysicalHealth_T1	0.84	13.01^*^	0.84	12.12^*^	0.84	12.33^*^	169.28	0.710
Neuroticism_T1			0.00	−0.01	−0.07	−0.88	83.41	0.001
Neuroticism_Dif					−0.14	−2.01	59.44	0.004
**NEOFFI-extraversion**								
PhysicalHealth_T1	0.84	13.01^*^	0.83	12.28^*^	0.84	12.53^*^	169.28	0.710
Extraversion_T1			0.04	0.56	0.06	0.93	83.96	0.001
Extraversion_Dif					0.11	1.63	58.23	0.011
**NEOFFI—consciousness**								
PhysicalHealth_T1	0.84	12.91^*^	0.83	12.53^*^	0.84	12.79^*^	166.71	0.710
Consciousness_T1			0.08	1.23	0.13	1.84	84.72	0.006
Consciousness_Dif					0.12	1.70	59.04	0.012

β, beta coefficient; *t*, Student t; *p*, significance level; ΔR^2^, increased explained variance; F, Snedecor F. “*” means the significance level *p* < 0.05.

Of the nine variables assessed, only Stress has a statistically significant effect. Focusing on this condition as a paradigm to explain the results of the table, in Step 1 it is observed that physical health component at baseline is introduced as a predictor of this component in the follow-up measure, explaining 70.56% of the variance (*F* = 169.28, *p* < 0.001; *β* = 0.84). In Step 2, Stress is introduced finding no statistically significant effect (*β* = −0.11; *t* = −1.37; *p* = 0.175), although a slight attenuation of the effect of the baseline physical component is observed (*β* = 0.77; *p* < 0.001). Finally, in Step 3, the variable Stress_Dif is introduced to previous predictors, expressing the increase in stress during the time of study. In this case, it is observed how high stress at baseline would adopt a predictive effect (*β* = −0.24; *t* = −2.83; *p* = 0.006) in conjunction with the increase in stress during the time of study (*β* = −0.22; *t* = −3.26; *p* = 0.002).

The same procedure has been conducted to explore the predictors of mental health perception (Table 4). Seven of the nine predictors have shown a significant effect. Only Emotional Control (*β_CE_* = −0.11; *β_CE_Dif_* = 0.02; *p* > 0.300) and Social Support (*β_SS_* = 0.07; *β_SS_Dif_* = 0.07; *p* > 0.445) have not shown any statistically significant effects. As in the case of physical health, stress has a high predictive effect, in fact greater (*β_St_* = −0.46; *β_St_Dif_* = −0.52; *p* < 0.001), and shows a comparable effect to the perception of mental health itself at baseline (*β_SM_* = 0.46; *t* = 3.58; *p* < 0.001). In other words, mental health perception at the beginning of the study explains 47.4% of the variance of health perception at the end of the study. The introduction of Stress at baseline has hardly any effect on the increased explained variance (*R^2^* = 0.005; 0.5%), but when it is introduced in conjunction with the increase in stress throughout the study (Step 3), the variance explained by both indicators is 21.2%.

**Table 4:** Regressions with mental health.

	Step 1		Step 2		Step 3			
	β	*t*	β	*t*	β	*t*	F	∆R^2^
**Stress**								
MentalHealth_T1	0.69	7.88^*^	0.57	3.50^*^	0.46	3.58^*^	62.14	0.474
Stress_T1			−0.14	−0.82	−0.46	−3.37^*^	31.26	0.005
Stress_Dif					−0.52	−6.77^*^	49.83	0.212
**Resilience**								
MentalHealth_T1	0.69	7.88^*^	0.70	6.13^*^	0.66	5.97^*^	62.14	0.474
Resilience_T1			−0.02	−0.19	0.11	0.94	30.65	0.000
Resilience_Dif					0.27	2.91^*^	25.51	0.059
**Emotional control**								
MentalHealth_T1	0.67	7.34^*^	0.65	6.94^*^	0.65	6.89^*^	53.85	0.457
EmotionalControl_T1			−0.10	−1.07	−0.11	−1.05	27.56	0.010
EmotionalControl_Dif					−0.02	−0.19	18.11	0.000
**Social support**								
MentalHealth_T1	0.69	7.88^*^	0.67	7.19^*^	0.67	7.15^*^	62.14	0.474
Social Support_T1			0.05	0.48	0.07	0.73	30.84	0.002
Social Support_Dif					0.07	0.77	20.63	0.005
**Adaptive coping**								
MentalHealth_T1	0.69	7.88^*^	0.69	7.32^*^	0.63	7.03^*^	62.14	0.474
Adaptive_T1			0.07	0.80	0.20	2.00^*^	31.23	0.005
Adaptive_Dif					0.25	2.54^*^	24.65	0.046
**Disadaptive coping**								
MentalHealth_T1	0.69	7.88^*^	0.69	5.66^*^	0.66	5.65^*^	62.14	0.474
Disadaptive_T1			−0.02	−0.02	−0.19	−1.45	30.62	0.000
Disadaptive_Dif					−0.29	−2.87^*^	25.33	0.058
**NEOFFI-neuroticism**								
MentalHealth_T1	0.69	7.88^*^	0.69	5.52^*^	0.63	5.85^*^	62.14	0.474
Neuroticism_T1			0.01	0.02	−0.23	−1.98^*^	30.62	0.000
Neuroticism_Dif					−0.43	−4.99^*^	35.89	0.143
**NEOFFI-extraversion**								
MentalHealth_T1	0.69	7.88^*^	0.76	7.39^*^	0.75	7.71^*^	62.14	0.474
Extraversion_T1			−0.13	−1.24	−0.07	−0.65	32.08	0.012
Extraversion_Dif					0.23	2.68^*^	25.73	0.050
**NEOFFI—consciousness**								
MentalHealth_T1	0.69	7.83^*^	0.63	7.05^*^	0.67	7.14^*^	61.29	0.474
Consciousness_T1			0.19	2.10^*^	0.23	2.42^*^	34.40	0.033
Consciousness_Dif					0.12	1.24	23.63	0.011

β, beta coefficient; *t*, Student t; *p*, significance level; ΔR2, increased explained variance; F, Snedecor F. “*” means the significance level *p* < 0.05.

A similar effect, but of lesser intensity, is observed in variables of Adaptive Coping and Neuroticism. In the case of Resilience (*β_Re_Dif_* = 0.27; *t* = 2.91; *p* = 0.005), Disadaptive Coping (*β_AD_Dif_* = −0.29; *t* = −2.87; *p* = 0.006) and Extraversion (*β_Re_Dif_* = 0.23; *t* = 2.68; *p* = 0.009), the effect on mental health at the end of the study is attributed to the observed change in these variables during the study process. Finally, Consciousness acquires a predictive effect at baseline (*β_Co_* = 0.19; *t* = 2.10; *p* = 0.039, *R^2^* = 0.033; Step 2), which increases slightly in Step 3 with the introduction of the change score (*β_Co_* = 0.23; *t* = 2.42; *p* = 0.018). However, the increase in explained variance is not significant (*R^2^* = 0.011; *F*_(1.66)_ = 1.54; *p* = 0.219).

### Predictive model of health status

Eight of the 19 explored variables correspond to health outcome variables: emotional distress (GHQ), health-related quality of life (physical and mental health summary components of SF-12), and health perception (five EVA variables). In order to assess and establish, where appropriate, a single indicator of the health construct, this set of variables was subjected to an Exploratory Factor Analysis (AFE), with a resulting factor solution for the baseline and follow-up time.

On the one hand, the AFE at baseline shows adequate convergence of the correlation matrix (KMO = 0.79; Bartlett’s Test *χ^2^* = 409.46, *p* < 0.001) with a single factorial solution (1st Eigenvalue = 4.46, 2nd Eigenvalue = 0.94) that explains a 55.77% of the variance, and with factor loads ranging from a minimum of 0.60 (mental health component of SF-12) to a maximum of.81 (EVA quality of life perception), all loads being positive except for GHQ, which has been negative (−0.80). This factorial solution is conceptually consistent, so it has been decided to accept as the sole indicator of health status (Health Outcome T1—HO-T1).

On the other hand, the same procedure was performed with the set of variables measured at the follow-up. The AFE also showed suitability for factoring the correlation matrix (KMO = 0.86; Bartlett’s Test *χ^2^* = 505.01, *p* < 0.001), with a two-factor factorial solution (1st Eigenvalue = 5.15, 2nd Eigenvalue = 1.05) that explained 64.43% and 13.21%, respectively. Since the eigenvalue of the first factor is almost five times greater than the second, it was decided to retain the solution of the first factor as an indicator. In this regard, factor loads resulting from retaining this first factor range from a minimum of 0.63 (mental health component of SF-12) to a maximum of.88 (EVA’s quality of life and health in the last month), with a factor load of −0.81 in GHQ. As in the previous case, a single factor (Health Outcome T2—HO-T2) is also accepted in the follow-up measurement, constructing a health outcome indicator from the factor solution. The correlation between both constructed factors (HO-T1 vs. HO-T2) is.79 (*p* < 0.001) and, given that they are two standardized variables, no differences are found in the averages of both assessment moments (*t* = 0.00, *p* = 1.00).

**Figure 1:**
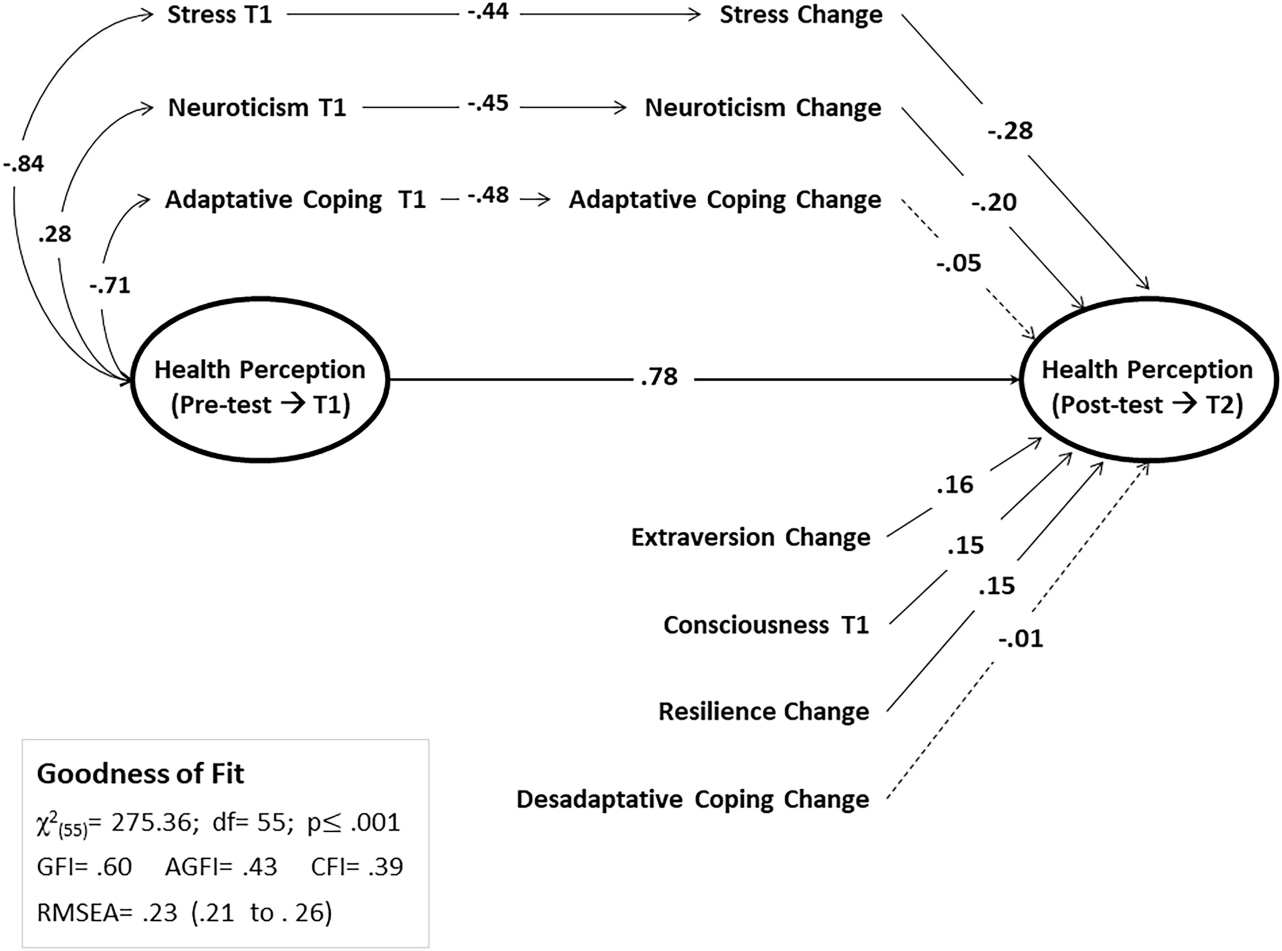
Path analysis on the predictive model of health perception.

Once health status indicators have been identified at both time periods (baseline and follow-up), and based on the information observed in previous sections, it has been decided to test a path model (path analysis) that would provide information on the variables that influence change in health perception in participants. The model that approaches closest to a satisfactory solution is represented in Figure 1, although the adjustment indexes do not sufficiently meet the adequacy criteria.

The model identifies participant’s baseline health perception as the principal predictor of health perception. This positive effect (*β_PS_* = 0.78; *p* < 0.001) implies that people with compromised health at the beginning of the study continue perceiving it as delicate once time has passed; and those who perceive their health status as appropriate at baseline continue perceiving it that way months later. Stress and Neuroticism have a noticeable, but not direct, effect on health status perception in the follow-up, and particularly, through the change that occurs in these variables over time (*β_St_* = −0.28 and *β_Ne_* = −0.20, both *p* < 0.001, respectively).

Let us assess the case of stress as a model for interpreting the result. Stress perception at baseline is negatively associated with health perception (*r* = −0.84), so that higher stress would be associated with poorer health perception. On the other hand, stress at baseline is also negatively associated with the change in stress through time (*β_St_* = −0.44, *p* < 0.001). Furthermore, this last variable (Stress_Dif) has a predictive effect on health perception in the follow-up (*β_St-Dif_* = −0.28, *p* < 0.001), indicating that the people who perceive less change in stress levels are those who perceive better health.

Other three variables show moderate but significant effects (*p* < 0.001) on health perception in the follow-up: Consciousness at baseline (*β_Co_* = 0.15; *p* = 0.003), the change in Extraversion (*β_Ex_* = 0.16; *p* = 0.001) and Resilience (*β_Re_* = 0.15; *p* = 0.002). Finally, this model does not identify a significant effect of coping on health perception, neither in the case of change in adaptive coping (*β_St_* = −0.05, *p* = 0.198) nor in disadaptive coping (*β_St_* = −0.01, *p* = 0.444).

## Discussion

The aim of this study was to explore a health global model that integrated the relationships between psychological variables involved in the oncological process through a longitudinal design. Results do not allow a conclusive model, but this study provides an approach to identify variables with a predictive effect over health perception of people with cancer.

In general, the sample showed moderate health perception levels. These results are significant if the severity of the illness is considered. Participants did not show an excellent health perception, but neither catastrophizing nor disturbing scores. When analyzing participants’ health scores over time, specifically, after a period of ~6 months, great changes were not found; scores were maintained in general. It should be noted that change scores showed some adaptive progression in the follow-up period. In addition, a decrease in aspects such as stress, emotional distress and neuroticism level was observed in participants. However, observed changes in the analyzed variables did not show certainty about the relevance of these changes in the scores. Actually, moderate effect sizes reflected some improvement on mean values but did not show a substantial recovery or improvement in scores. These results could be explained since most of the participants of the study were attending AECC, where they had received and/or are receiving psychological assistance (91.4% of them). Despite causal relationships cannot be concluded, it is noteworthy that health status in participants was not as poor as expected, especially considering the severity and the characteristics of the disease. Cancer can be a very stressful illness that threatens patients’ well-being and physical and psychological quality of life. At this respect, psychological interventions in oncological contexts acquire great relevance in the improvement of active and adaptive coping and the development of resources for facing the disease (Lim et al., 2014; de la Torre-Luque et al., 2016).

Precisely, this study aimed to explore the predictive effects of certain psychological variables on cancer patients´ general health perception. Health status was analyzed through two main indicators: physical and mental health. On the one hand, physical health perception was only significantly influenced by stress perception. However, the direction of this influence should be discussed. Higher stress was related to poorer physical health perception. And when stress levels were reduced, physical health perception was increased. Future research should explore deeply which factor has a predictive effect; that is, if high stress at baseline predicts worse physical health or, conversely, if lower perception of physical health increases stress perception in cancer patients.

In this study, it is noticeable that stress at baseline did not show any statistically significant association with physical health; but it showed a significant connection with it through the change in the follow-up (Stress_Dif). Subsequently, stress perception was considered as a predictor variable; nevertheless, deterioration in physical health perception could be also increasing stress levels. As mentioned before, cancer is a life-threatening disease that can lead to the deterioration of patients´ health and quality of life, causing impairments in daily life (Merluzzi et al., 2018). As consequence, patients´ can experience great levels of stress, depression and anxiety, associated to different disease-related aspects (oncological treatment, uncertainty about the prognosis, physical consequences, etc.; Seib et al., 2018). On the other side, as mentioned before, participants of this study could have reduced their level of stress due to the fact that they were attending psychological therapy at AECC. They could have acquired adaptive coping strategies and performed emotional regulation strategies that would have contribute to the reduction of stress during the disease and improve their level of physical health perception (Lim et al., 2014).

On the other hand, mental health perception was influenced by almost all the psychological variables that were analyzed. For instance, variables of Stress, Neuroticism and Adaptive Coping had a significant effect on mental health, particularly in the change observed in the follow-up period. Furthermore, Consciousness at baseline, Resilience and Extraversion in their change scores showed an indirect effect over mental health perception. These results are in accordance with other studies, which suggest the positive influence of variables such as resilience and certain personality features on cancer patients´ health (Bogg and Roberts, 2013; Min et al., 2013; Hoerger et al., 2016; Macía et al., 2020a). This evidence highlights the importance of empowering the person in acquiring resilience resources for facing the disease, rather than developing specific coping strategies. Psychological interventions should emphasize the increase of aspects such as resilience and extraversion in patients, through specific techniques focused in group-based sharing processes, resilience building interventions (increase of like self-esteem, cognitive flexibility, connectedness with significant others), development of therapeutic alliance and confidence, etc. (Solano et al., 2016; Reynolds, 2019; Macía et al., 2020b,c).

Conversely, Emotional Control and Social Support did not show any influence over mental health perception. These results contradict what other studies have found, which have considered the relevance of high perceived social support and emotional expression (opposite to emotional control) as factors related to positive health outcomes in cancer (Durá et al., 2010; Costa-Requena et al., 2014; Li et al., 2015; Macía et al., 2020b). In particular, absence of effect of social support over mental health is remarkable in this study. Literature suggests that it has a protective effect on mental health (Mishra and Saranath, 2019). Perceived social support in cancer patients prevents from developing symptoms of emotional distress that lead to psychological negative consequences (Thompson et al., 2017). Results in this study could be interpreted considering that participants were contacts of the Association (AECC). Therefore, they might be characterized by a social profile that would result in high test scores, leading to an absence of discrimination in social support scale scores.

## Study limitations

There are some limitations in this study. Firstly, the sample size limits the generalization of the results. Considering the characteristics of the disease, collecting sample in the follow-up period becomes challenging, due to many aspects such as those related to the requirements of the treatment, the worsen of the health status, among others. Secondly, the variability of the sample should be reduced. Future research should attempt to increase homogeneity regarding the stage of the disease, type of tumor, sociodemographic characteristics of the sample, etc.

Besides, future investigations could explore more deeply the predictive effects of psychological variables on health perception in further follow-up periods. Probably due to the limited sample size and the large number of variables analyzed, the conducted path analysis does not fit adequacy criteria sufficiently, although it provides information about the underlying relationships among the assessed variables. It would be interesting to perform this integrative model with an increased cancer sample, even conduct comparisons analysis with a normative healthy group.

## Clinical implications

This evidence has a great relevance for clinicians who work in therapeutic contexts in the oncological area. Findings in this study provide information about which psychological variables contribute more to the increase of general health perception in cancer patients. Specifically, the reduction of stress and neuroticism, as well as the increase in resilience and some personality traits (such as extraversion and consciousness) seem to be positive aspects influencing health perception in cancer patients. This information could be useful for guiding the design of tailored and personalized psychotherapeutic interventions adapted to each person, with the aim of increasing adaptation to disease.

## Conclusion

Results in this study allow an approach to a global health model that integrates and explores associations between psychological variables related to cancer disease process. Although the final model does not allow a conclusive interpretation, it provides a representation of the underlying relationships and the predictive effects of certain psychological aspects on the general health perception in people with cancer.

Findings show that stress has a potential influence on cancer patients´ health perception. However, this influence also occurs in the opposite direction, as health status is also influencing stress perception in participants. In addition, results in this study reflect that general health status in participants is not as poor as expected, although emotional distress is also present. These results might be explained since participants were contacts of the Association (AECC), where a great majority of the participants were receiving psychotherapeutic care or support. It would be interesting to conduct further research focused on the specific effects of psychotherapy in cancer patients. Furthermore, evidence in this study remarks the importance of developing psychological interventions focused on the increase of extraversion and resilience in people with cancer.

This study has been developed on the basis of the concept of Person-Centered Care in the oncological area. From this conceptualization, the person is the focus of the interventions and plays an active role in own health decisions. This concept emphasizes the significance of considering personal psychological characteristics in order to optimize psychotherapeutic prescriptions. Existing literature has evidenced the influence of some personal variables such as resilience, emotional control, coping, etc. on cancer patients´ psychological well-being, mental health and quality of life. However, there was a lack in the literature about the explanation of how all these variables are related. In the present study, we conducted an integrated research project where assessing some psychological variables related to adjustment to cancer disease all together: resilience, coping strategies, emotional control, perceived stress, social support, personality, affectivity, and other outcome variables such as mental health and quality of life.

This study has overcome literature gaps regarding the underlying mechanisms that explain the specific interaction and the relationship between psychological variables and health in people with cancer through and integrating and global model. Definitely, we found this information useful in order to design and guide specific therapeutic interventions, which could encourage aspects such as emotional expression, resilience and adaptive coping in cancer patients.

## Data availability statement

The dataset generated for this study can be found in the Figshare data repository: doi: 10.6084/m9.figshare.13704394.

## Ethics statement

The protocol was approved by the University of Deusto Research Ethics Committee (ETK-19/19-20). The patients/participants provided their written informed consent to participate in this study.

## Author contributions

PM and SG has conducted the statistical analysis and the drafting of the manuscript and worked on the acceptance of the final paper. MB has participated in the planning and design of the study and worked on the final drafting of the manuscript, and is responsible for the coordination of the field study. NI has worked in the drafting and on the acceptance of the final paper. II has conducted the statistical analysis and the drafting of the manuscript, worked on the acceptance of the final paper, and is the Principal Investigator of the “Evaluación, Clínica y Salud” research team of Deusto University. All authors contributed to the article and approved the submitted version.

## Conflict of interest

The authors declare that the research was conducted in the absence of any commercial or financial relationships that could be construed as a potential conflict of interest.

## Publisher’s note

All claims expressed in this article are solely those of the authors and do not necessarily represent those of their affiliated organizations, or those of the publisher, the editors and the reviewers. Any product that may be evaluated in this article, or claim that may be made by its manufacturer, is not guaranteed or endorsed by the publisher.
